# Genomic DNA Methylation Changes in Response to Folic Acid Supplementation in a Population-Based Intervention Study among Women of Reproductive Age

**DOI:** 10.1371/journal.pone.0028144

**Published:** 2011-12-07

**Authors:** Krista S. Crider, Eoin P. Quinlivan, Robert J. Berry, Ling Hao, Zhu Li, David Maneval, Thomas P. Yang, Sonja A. Rasmussen, Quanhe Yang, Jiang-Hui Zhu, Dale J. Hu, Lynn B. Bailey

**Affiliations:** 1 Division of Birth Defects and Developmental Disabilities, National Center on Birth Defects and Developmental Disabilities, Centers for Disease Control and Prevention, Atlanta, Georgia, United States of America; 2 Biomedical Mass Spectrometry Laboratory, General Clinical Research Center, University of Florida, Gainesville, Florida, United States of America; 3 Peking University Health Science Center, Beijing, China; 4 The Food Science and Human Nutrition Department, University of Florida, Gainesville, Florida, United States of America; 5 The Center for Epigenetics, Division of Pediatric Genetics, Department of Biochemistry and Molecular Biology, University Florida College of Medicine, Gainesville, Florida, United States of America; Chinese Academy of Science, China

## Abstract

Folate is a source of one-carbons necessary for DNA methylation, a critical epigenetic modification necessary for genomic structure and function. The use of supplemental folic acid is widespread however; the potential influence on DNA methylation is unclear. We measured global DNA methylation using DNA extracted from samples from a population-based, double-blind randomized trial of folic acid supplementation (100, 400, 4000 µg per day) taken for 6 months; including a 3 month post-supplementation sample. We observed no changes in global DNA methylation in response to up to 4,000 µg/day for 6 months supplementation in DNA extracted from uncoagulated blood (approximates circulating blood). However, when DNA methylation was determined in coagulated samples from the same individuals at the same time, significant time, dose, and *MTHFR* genotype-dependent changes were observed. The baseline level of DNA methylation was the same for uncoagulated and coagulated samples; marked differences between sample types were observed only after intervention. In DNA from coagulated blood, DNA methylation decreased (−14%; *P*<0.001) after 1 month of supplementation and 3 months after supplement withdrawal, methylation decreased an additional 23% (*P*<0.001) with significant variation among individuals (max+17%; min-94%). Decreases in methylation of ≥25% (vs. <25%) after discontinuation of supplementation were strongly associated with genotype: *MTHFR* CC vs. TT (adjusted odds ratio [aOR] 12.9, 95%CI 6.4, 26.0). The unexpected difference in DNA methylation between DNA extracted from coagulated and uncoagulated samples in response to folic acid supplementation is an important finding for evaluating use of folic acid and investigating the potential effects of folic acid supplementation on coagulation.

## Introduction

DNA methylation is an epigenetic modification required for normal gene expression and the chromosomal integrity essential for embryogenesis [1], [2], [3]. Folate in the form of N-5-methyltetrahydrofolate (5-methylTHF) provides the methyl groups for the formation of *S*-adenosylmethionine (SAM), the body's universal methyl donor required for DNA methylation. Common single-nucleotide genetic variants associated with folate metabolism might have a significant effect on folate-dependent, one-carbon transfer reactions. One key polymorphism is the 677C→T variant of the methylenetetrahydrofolate reductase (*MTHFR*) gene, responsible for the synthesis of 5-methylTHF. When coupled with low folate status, the *MTHFR* 677C→T variant has been reported to be associated with elevated homocysteine concentrations; reduced global DNA methylation; and a variation in the risk for many disorders, including neural tube defects (NTDs) and venous thrombosis [4], [5], [6]. Among populations with high prevalences of the *MTHFR* T allele and vitamin B12 deficiency, such as the population in North China in this study [7], [8], it has been suggested that the selective advantage of the T allele in these groups might be pregnancy-related, such as resistance to anemia and hemorrhage during childbirth [9]. The *MTHFR* 677C→T variant may be associated with a diversion of available methyl groups from the DNA methylation pathway towards the DNA synthesis pathway and moderate the anemia due to a vitamin B12 deficiency [9], [10], [11].

Data from small-scale human metabolic studies have provided evidence that folate depletion and repletion can affect the global methylation status of DNA from peripheral blood cells [12], [13], [14]. However, there have been no previous population-based studies that provide data that describe normal DNA methylation and/or how it might be affected by initiation of folic acid supplementation and subsequent withdrawal of supplementation, differences in folic acid dose, and/or *MTHFR* genotype.

Based on evidence that periconceptional folic acid significantly reduces NTD risk [15], folic acid is recommended for NTD prevention in doses of 400 or 4,000 µg/day for NTD occurrence and recurrence, respectively, for women of childbearing age [16]. Mandatory folic acid fortification was implemented in the United States, based on the evidence that folic acid reduces NTD risk and provides ∼100 µg/day [17]. A large-scale, randomized, population-based folic acid intervention trial was conducted in Northern China for the purpose of selecting a specific folic acid dosing regimen for future NTD prevention programs in which the folate status responses to 100-, 400-, and 4,000-µg/day folic acid doses were compared as well as the interaction of folic acid dose with *MTHFR* genotype [7], [8]. In the present study, samples from the previous study were utilized to assess whether there were changes in global DNA methylation in response to folic acid supplementation among women of reproductive age not exposed to dietary sources of folic acid or folic acid food fortification. The possibility of adverse effects of folic acid use through changes in DNA methylation has been suggested [18]. However, the potential effects of the initiation and termination of folic acid supplement use on global DNA methylation is unknown.

This study's objectives were to determine what, if any, global DNA methylation changes accompany folic acid supplementation and withdrawal and if the changes are time-, dose-, or genotype-dependent.

## Materials and Methods

### Ethics Statement

All participants provided oral informed consent which was documented with a signature of the consenting investigator as was culturally appropriate for the research setting and was permissible as the study posed no more than minimal risk of harm to the participant and involved no procedure for which written consent was normally required outside of a research setting. The study and consent procedures (including a waiver for the documentation of informed consent as set forth in 45CFR46.117(c)) were approved by the Centers for Disease Control and Prevention Institutional Review Board and the Ethical Committee on Biomedical Research Involving Human Subjects of the Health Science Center, Peking University.

### Setting and inclusion criteria for participants

**Figure 1:**
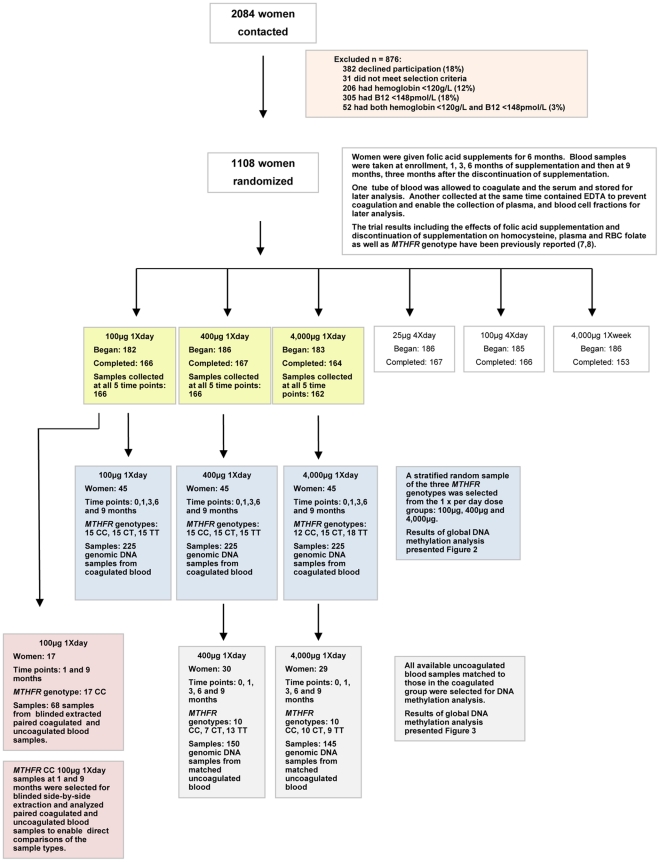
Study design and sample selection.

Women in the study were recruited from six townships of Xianghe County, Hebei Province, in Northern China, near Beijing (the trial is registered at clinicaltrials.gov: NCT00207558). As previously described, to be eligible for the study, women were required to live in the township; have a child 2–4 years of age; not be pregnant or breastfeeding; have no plans to become pregnant during the subsequent 9 months; be using an intrauterine device for contraception; have no chronic diseases; have had no folic acid supplement use within the last 3 months; and have no current prescription medication use [7]. Participants were screened at enrollment and excluded if they were either vitamin B12-deficient (plasma vitamin B12<148 pmol/L) or anemic (hemoglobin <120 g/L) (Fig. 1). Since folic acid-fortified foods were not available in China, study participants were not exposed to dietary sources of folic acid.

### Study design

Eligible participants were randomized into three daily folic acid treatment groups (100 µg, 400 µg, and 4,000 µg) which were taken for 6 months. Participants had blood samples drawn at enrollment; after 1, 3, and 6 months of folic acid supplementation; and 3 months after withdrawal of supplementation. The repeated measures design of the study allowed each participant to serve as her own control; therefore, a placebo group was not included. A double-blind randomization was used to assign equal numbers of women to each of the folic acid intervention groups as described previously [7].

### Blood collection procedures

Fasting blood samples were collected at the time of enrollment, and at the end of 1, 3, 6, and 9 months using standard phlebotomy techniques. For each participant, blood was collected into 7 ml tubes with EDTA and 3 ml tubes without any anticoagulants (Vacutainer; Becton Dickinson, Franklin, NJ). After centrifugation, plasma from the 7 ml tubes and sera from the 3 ml tubes were removed. Plasma was separated by centrifugation at 4°C, 2000× g for 15 min, and frozen at −20°C within 1 hour of collection. The serum was separated by the same centrifugation after 1–2 hour of coagulation at room temperature. All blood cells and clots were then frozen at −20°C and transported on dry ice to the central laboratory of the Peking University Health Science Center in China and stored at −70°C until sent by air courier on dry ice to the University of Florida in the United States for DNA extraction and analysis.

### Genomic DNA extraction and genotyping

Initially, only blood clots from coagulated blood were shipped from China and available for genomic DNA analysis. Using a commercially available blood DNA purification kit (Gentra Puregene, Qiagen, Valencia, CA), genomic DNA was extracted from stored frozen blood clots according to the manufacturer's instructions. Thawed clots were digested with proteinase K after dispersal, either by centrifugation through perforated inserts (Clotspin, Qiagen) or by mincing with paired, curved scalpel blades in plastic weigh boats. An alternative automated method (Maxwell 16, blood kit, tissue protocol, Promega, Madison, WI) produced equivalent DNA yield and results (data not shown). Samples were hydrated in TE buffer, and DNA concentration was determined by fluorescent dye binding assay (Quant-iT, Invitrogen, Carlsbad, CA) and adjusted to 20 ng/µl.

*MTHFR* 677 C→T genotyping procedures were described previously [8]. Samples were genotyped by sequential participant number and researchers were blinded to dose group and all participant data. Briefly, *MTHFR* 677 C→T genotypes were determined on 3-µl aliquots of genomic DNA by dynamic allele-specific hybridization [19]. Analyses were performed with dFOLD and DYNASCORE v. 0.65 software (DynaMetrix Limited, Stockholm, Sweden; Internet: http://www.dynametrix-ltd.com) and genotypes were confirmed for 15% of randomly selected samples with probes for the alternate allele [20].

### Sample selection

To assess the effects of folic acid dose, length of exposure, withdrawal, and *MTHFR* 677 C→T genotype on global DNA methylation, a stratified random sample of 135 individuals with 5 blood samples was selected for analysis at each time point (at enrollment; after 1, 3, and 6 months of supplementation; and 3 months after withdrawal of supplementation; samples *n* = 675) (Fig. 1). From each daily dose group (100 µg, 400 µg, and 4,000 µg), 45 participants were chosen with the intent to select 15 from within each group to equally represent each of the three *MTHFR* genotypes (CC, CT, and TT). As a result of the lower prevalence of the *MTHFR* CC genotype, 15 CC samples were not available from the 4,000 µg/day dose group; therefore, 12 CC samples were selected, and 3 additional TT samples were added to attain the *n* = 45 for this subgroup. Sampling was accounted for in the analysis through weighting and adjusting.

Initially, only blood clots from coagulated blood were available for genomic DNA analysis. After the global methylation analysis of the 675 samples from coagulated blood was completed, red blood cell (RBC) samples from uncoagulated blood from the same individuals were shipped frozen on dry ice from China to the United States for additional DNA methylation analysis. These samples were used for routine DNA methylation confirmatory testing, at which time it was discovered that there were inconsistencies between the DNA extracted from coagulated and uncoagulated blood samples from the same individuals. Therefore, additional liquid chromatography-mass spectrometry (LC-MS/MS) analyses using both sample types were preformed. The first confirmatory testing was a blinded side-by-side extraction and LC-MS/MS analysis of 1- months and 9 months samples from the 100 µg/day MTHFR CC supplementation group (*n* = 68) (Fig. 1). Samples for this analysis were selected based on data obtained in previously analyzed DNA extracted from clots that showed significant decreases in DNA methylation in this group. The second was a blinded study designed to completely replicate the DNA methylation analysis of coagulated samples for the 400 µg/day (*n* = 30) and 4,000 µg/day (*n* = 29) dose groups using uncoagulated blood from the same participants for which matched blood samples were available (total sample *n* = 295) (Fig. 1).

### LC-MS/MS analysis of 5-methyl-deoxycytidine (MdCyt)

Prior to digestion, internal standards (biosynthetic [^15^N_3_]dCyt and [^15^N_3_]MdCyt) were added to 0.5 µg of DNA sample as described elsewhere [21]. DNA was then digested to deoxynucleosides using the procedure of Quinlivan and Gregory [22]. Digests were chromatographed [21] on a Luna 3µ C18 column (50×3.0 mm; Phenomenex, Torrence, CA) using an ammonium acetate:methanol gradient. Samples were analyzed in ESI mode using a triple quadrupole mass spectrometer. Scanning was performed in selected reaction monitoring (SRM) mode, monitoring the mass-to-charge (m/z) transitions of dCyt, 228→112; [^15^N_3_]dCyt, 231→115; MdCyt, 242→126; and [^15^N_3_]MdCyt, 245→129. Chromatograms were analyzed using the Xcalibur software package (Thermo; Version 2.0). Concentrations of dCyt and MdCyt were determined by comparing the analyte/internal standard (M+0/M+3) peak area ratio to standard curves. The ratio of MdCyt to total dCyt was used to calculate %MdCyt, i.e., %MdCyt = MdCyt/(dCyt+MdCyt)×100. Inter- and intra-assay variation (relative standard deviation; n = 6) was <2.5%. Additional details of the methodology have been published elsewhere [21], [22]. The operator (EPQ) was blinded with respect to sample dose group or genotype. Samples were analyzed in a single continuous run, ordered only by sequential participant numbers. To minimize any error due to position in the analysis cue, samples from the serial blood draws from each participant were assayed in juxtaposition. Two highly correlated replicate assays were run on coagulated samples: a complete replication of the enrollment readings and a two-thirds replication of the 6 and 9 months time points. Independent analysis of each data set led to the same interpretation of the results.

### Methyl-acceptance assays

To confirm the results of global DNA methylation analysis by LC-MS/MS, [^3^H] methyl-acceptance assays were performed on representative samples that showed either large or moderate changes in methylation by LC-MS/MS in DNA extracted from coagulated blood. Using a modification of the method of Balaghi and Wagnerm [23], DNA methylation was determined as we have previously described [13]. The ability of cytosine in DNA to accept and incorporate [^3^H] methyl groups in the presence of SssI prokaryotic methylase is inversely related to the level of DNA methylation.

### Statistical analysis

Arithmetic means and standard deviations were calculated to show the participant characteristics and response to folic acid supplementation and withdrawal. General linear models for repeated measures were used to determine factor associated with DNA methylation level and to obtain estimated means and 95% confidence intervals (CIs) to allow for correlation between the measurements at sequential time points and provide and examine the effects of both folic acid dose and *MTHFR* genotype and the interaction between dose and genotype. So that the results could be generalized to the population from which the study participants were selected, the model was weighted to account for sampling. The percent DNA methylation and enrollment RBC folate concentrations were log-transformed to normalize the data for modeling purposes only. The models used folic acid dose, *MTHFR* genotype, age, body mass index (BMI), and enrollment RBC folate concentration as covariates. In coagulated samples, the folic acid intervention and withdrawal were modeled separately to allow for the differential effects of covariates on the different exposures as specified.

Logistic regression was used to determine factors associated with a ≥25% decrease or a ≥50% decrease in DNA methylation after withdrawal of folic acid supplementation in coagulated samples. The models included folic acid dose (using the 4,000-µg/day dose as referent), *MTHFR* genotype (TT genotype as referent), age, and baseline RBC folate concentration. SAS-JMP Genomic and SPSS statistical packages were used to analyze the data.

Power analysis based on the SD at enrollment in coagulated samples indicated that there was >80% power to detect differences of ≥5% with five samples. Given the larger SD after the intervention in coagulated samples, power analysis indicated >90% power to detect differences of >15% using 12 (the smallest coagulated subgroup sample size) samples per group.

## Results

### Demographic, vitamin B12, and hemoglobin data at baseline in the subsample used for DNA methylation analysis

The mean age of the women was 30.4±4 years and did not differ between treatment groups (Table 1). There were no differences in the average BMI, plasma vitamin B12, or hemoglobin concentrations for participants in the three folic acid supplementation groups (Table 1).

**Table 1:** Participant characteristics and response to folic acid supplementation and termination of supplementation.

	Folic acid dosage					
	100 µg/d		400 µg/d		4,000 µg/d	
	Mean	SD	Mean	SD	Mean	SD
Age	30.6	4.1	30.7	3.7	29.9	4.4
Body mass index	23.5	3.9	24.1	4.9	24.2	4.3
B12 (pmol/l) screening	287.2	122.4	277.0	96.9	287.2	99.2
Plasma folate (nmol/L) screening	10.5	5.2	10.0	4.9	9.9	4.4
1 month	19.5	8.1	31.4	12.2	103.0	129.3
3 months	23.4	12.1	39.2	16.3	92.2	122.8
6 months	36.7	70.1	40.9	19.8	56.3	34.3
3 months withdrawal (9 mo)	16.2	5.5	17.4	6.3	19.2	5.8
Red blood cell folate (nmol/L) screening	685.2	292.8	669.7	365.6	685.1	309.7
1 month	706.2	360.9	785.2	292.5	1031.0	470.9
3 months	712.5	245.6	916.6	289.1	1543.8	707.0
6 months	821.9	363.8	1147.8	546.3	1608.7	835.1
3 months withdrawal (9 mo)	653.2	259.1	726.0	298.3	821.4	337.8
Plasma homocysteine (Hcy)(µmol/L) screening	8.2	4.4	8.9	6.5	8.9	5.4
1 month	8.9	4.6	8.7	6.0	7.7	3.3
3 months	7.0	3.6	6.5	4.2	6.2	3.0
6 months	6.8	2.7	7.1	5.6	7.4	4.5
3 months withdrawal	8.1	4.0	8.0	5.2	7.7	4.1
Hemoglobin (Hgb) (g/L) screening	138.3	9.6	135.7	9.0	133.6	8.1
6 months	146.8	15.2	147.0	13.5	148.0	13.0
3 months withdrawal (9 mo)	136.4	10.9	136.9	10.9	136.0	10.6

### Blood folate and homocysteine concentrations in the subsample used for DNA methylation analysis

Detailed analysis of the plasma and RBC folate and homocysteine response and response by *MTHFR* genotype are published elsewhere [7], [8]. The effects of folic acid supplementation and withdrawal on folate and homocysteine concentrations for the stratified random sample use in this study are presented in Table 1. Blood folate concentrations increased after initiation of folic acid supplementation in each group (trend 4,000 µg>400 µg>100 µg) with a sharp decrease after 3 months of folic acid withdrawal.

### Determination of global DNA methylation changes associated with initiation of folic acid supplementation—coagulated blood

**Figure 2:**
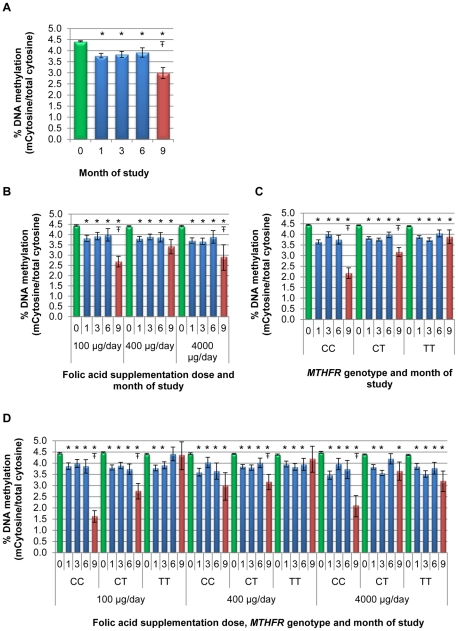
Global DNA methylation changes in response to folic acid supplementation and withdrawal among women of reproductive age- Coagulated Blood. Enrollment % DNA methylation (%MdCyt/total cytosine) is shown in green, DNA methylation level during the supplementation trial is shown in blue and DNA methylation level after the discontinuation of supplementation (withdrawal) is shown in red. **A.** Overall estimated mean %MdCyt and 95% Confidence Intervals (CI) were generated from a general linear model of repeated measure model (GLMRM) containing; folic acid dose, *MTHFR* genotype, age, BMI, enrollment RBC and time (0,1,3,6,9 months data) weighted to adjust for sampling. The model showed a significant interaction of folic acid dose and *MTHFR* genotype (P<0.001). **B.** Estimated mean %MdCyt and 95% CI were generated from a GLMRM stratified by folic acid dose; adjusting for *MTHFR* genotype age, BMI, enrollment RBC and time and weighted to adjust for sampling. **C.** Estimated mean %MdCyt and 95% CI were generated from a GLMRM stratified by *MTHFR* genotype; adjusting for folic acid dose, age, BMI, enrollment RBC and time and weighted to adjust for sampling. D. Estimated mean %MdCyt and 95% CI were generated from a GLMRM stratified by *MTHFR* genotype and folic acid dose; adjusting for age, BMI, enrollment RBC and time and weighted to adjust for sampling. * Significant difference compared to enrollment p<0.01 –^Ŧ^ Significant difference between the DNA methylation level at the end of the supplementation trial (6 months) and after the discontinuation of supplementation (9 months) p<0.01.

Significant changes in DNA methylation (determined by LC-MS/MS expressed as the percentage of 5-methyl-deoxycytidine [%MdCyt] in total genomic deoxycytidine [MdCty plus unmethylated dCyt]) were observed with initiation of folic acid supplementation for all three dose groups and *MTHFR* genotypes (Fig. 2). At enrollment, there was minimal intra-individual variation in global DNA methylation (unadjusted average %MdCyt = 4.42 [SD+/−0.12], min. 4.12, max. 4.74), comparable to previous estimates of DNA methylation levels in a variety of mammalian tissues [12], [24]. There was a decrease in DNA methylation in all participants after 1 month. After 1, 3, and 6 months, there was increased intra-individual variation in global DNA methylation (after 1 months unadjusted mean: %MdCyt = 3.83 [SD+/−0.42], min. 1.06, max. 4.33; after 3 months unadjusted mean: %MdCyt = 3.83 [SD+/−0.40] min. 1.12, max. 4.55; after 6 months of supplementation unadjusted mean: %MdCyt = 4.03 [SD+/−0.72], min. 0.65, max. 5.25). At the end of 6 months, 27% of individuals with a *MTHFR* T allele showed a ≥5% increase in methylation (max+19.5%); however, no increases were observed for individuals with the *MTHFR* CC genotype. In general, variability in DNA methylation levels increased with increasing folic acid dosage, time and presence of the *MTHFR* C allele.

In response to 1 month of folic acid supplementation, an overall 14% decrease (*P*<0.0001) was observed in global DNA methylation when using a general linear model for repeated measures (GLMRM) adjusted for both potential confounders and weighted to allow for inference to the population (Fig. 2 and supplemental Table S1). The models showed that the decrease in DNA methylation at 1 month was independent of folic acid dose (*P*>0.5) and *MTHFR* genotype (*P*>0.3).

When stratified by folic acid dose and genotype, a similar pattern of DNA methylation decrease after initial of supplementation was observed throughout the course of the intervention (Fig. 2). An interaction between dose and genotype with DNA methylation (*P*<0.001) was found. The estimated means of the methylation level at 6 months in the 100 µg/day *MTHFR* TT genotype group and in the 4,000 µg/day *MTHFR* CT group were not significantly different from the estimated mean at enrollment (Fig. 2).

### Global DNA methylation changes associated with the withdrawal of folic acid supplementation—coagulated blood

The change in DNA methylation level observed between enrollment and 3 months after the withdrawal of folic acid supplementation varied from a 94% decrease to a 17% increase (unadjusted mean %MdCyt 3.46 [SD+/−1.17], min. 0.24, max. 4.9). A partial-replicate study employing identical independent procedures yielded essentially identical results (data not shown).

An additional overall 23% decrease (*P*<0.001) in percent global DNA methylation was observed in response to folic acid withdrawal after supplementation using an overall weighted, adjusted general linear model for repeated measures (Fig. 2 and supplemental Table S1). Differences in magnitude of the decrease between dose groups were indicated by stratifying by folic acid dose (Fig. 2). There was a clear dose response by *MTHFR* genotype, with the greatest decrease in DNA methylation observed among the *MTHFR* CC (41.7%), CT (20.1%), and TT (3.6%-non significant decrease). The genotype-dose combinations with the largest decrease in % methylation were among individuals with the *MTHFR* CC genotype receiving 100 µg/day (57.7%) , with a small, non-significant mean increase in DNA methylation among the 400 µg/day *MTHFR* TT group (6.6%). The 100 µg/day group showed a clear dose-response, with the largest drop observed for the CC genotype group (57.7%), followed by the CT (25.9%) and TT (1.2%) groups compared to the % methylation after 6 months of supplementation. Among those receiving higher doses, the CT genotype more closely resembled the CC genotype in the 400 µg/day and the TT 4000 µg/day groups. The model revealed an interaction of dose and genotype (P<0.001) for the withdrawal phase of the trial.

Following folic acid withdrawal, there was a strong association between a decrease in DNA methylation of ≥25% (vs. <25%) and genotype: *MTHFR* CC vs. TT (adjusted odds ratio [aOR] 12.9, 95% CI 6.4, 26.0) and CT vs. TT (OR 2.8, 95% CI 1.6, 5.1), as seen in a logistic regression model adjusted for age, baseline RBC, and folic acid dosage. Similar trends were seen if a decrease of ≥50% in DNA methylation (vs. <50%) was used in the model, with associations noted with genotype (*MTHFR* CC vs. TT [aOR 20.1, 95% CI 7.8, 51.3) and CT vs. TT [aOR 2.0, 95% CI 0.8, 5.2]).

### [^3^H] Methyl-acceptance assay—Coagulated blood

Pairs of samples (n = 7) were also analyzed by the [^3^H] methyl-acceptance assay to confirm the LC-MS/MS, data: 4 pairs with large decreases between enrollment and withdrawal ranging from −89% to −79% by LC MS/MS and 3 pairs with moderate changes (−4% to+ 0.9%). The results indicated that samples with moderate changes in DNA methylation by LC-MS/MS (mean −1%, SD 2.2) also showed moderate changes when analyzed using the methyl-acceptance assay (mean 4%, SD 24), while those with large decreases in percent DNA methylation by the LC-MS/MS (mean = −86%, SD 5) assay, were also found to have large increases in methyl-acceptance (mean = 94%; SD 3) (indicating large decreases in methylation).

### Determination of global DNA methylation changes associated with initiation of folic acid supplementation and withdrawal of supplementation―Uncoagulated blood

**Figure 3:**
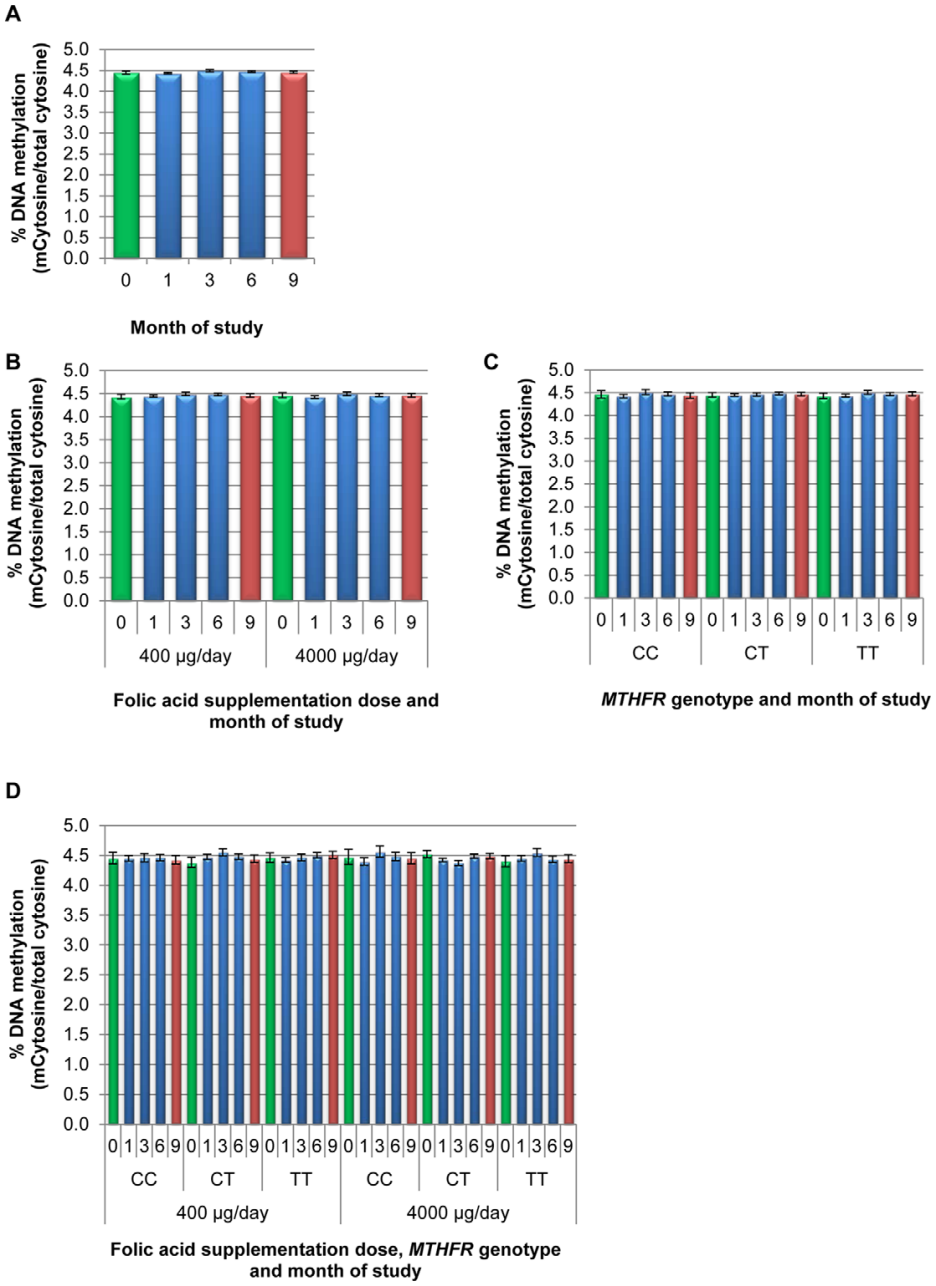
Global DNA methylation changes in response to folic acid supplementation and withdrawal among women of reproductive age- Uncoagulated Blood. Enrollment % DNA methylation (%MdCyt/total cytosine) is shown in green, DNA methylation level during the supplementation trial is shown in blue and DNA methylation level after the discontinuation of supplementation (withdrawal) is shown in red. **A.** Overall estimated mean %MdCyt and 95% Confidence Intervals (CI) were generated from a general linear model of repeated measure model (GLMRM) containing; folic acid dose, *MTHFR* genotype, age, BMI, enrollment RBC and time (0,1,3,6,9 months data) weighted to adjust for sampling. GLMRM modeling shows no significant effect of the *MTHFR* genotype or folic acid dose or an interaction of dose and genotype (all P>0.8). **B.** Estimated mean %MdCyt and 95% CI were generated from a GLMRM stratified by folic acid dose; adjusting for *MTHFR* genotype age, BMI, enrollment RBC and time and weighted to adjust for sampling. **C.** Estimated mean %MdCyt and 95% CI were generated from a GLMRM stratified by *MTHFR* genotype; adjusting for folic acid dose, age, BMI, enrollment RBC and time and weighted to adjust for sampling. D. Estimated mean %MdCyt and 95% CI were generated from a GLMRM stratified by *MTHFR* genotype and folic acid dose; adjusting for age, BMI, enrollment RBC and time and weighted to adjust for sampling. All pairwise comparisons between enrollment and months of supplementation and withdrawal were non-significant and showed a <+/− 3% change which is within the variability of the LC-MS/MS assay. All subjects included in this table are a subset of coagulated results in Figure 2.

In uncoagulated samples, the %MdCyt was very similar to the baseline levels in the DNA from coagulated blood samples (%MdCyt = 4.42 [SD+/−0.12] coagulated samples vs. mean %MdCyt = 4.46 [SD+/−0.15] uncoagulated samples). At baseline there were no significant difference between the three MTHFR genotypes (ANOVA, *P*>0.9 for all comparisons, mean %MdCyt = 4.46 [SD+/−0.15]). There was no increase in the variation in methylation level between the intervention groups at 9 months %MdCyt = 4.46 [SD+/−0.15]. Using a general linear model for repeated measures, there were no changes in mean methylation levels in response to folic acid supplementation or the termination of supplementation in the 400 µg/day and 4,000 µg/day group doses or within the 400 µg/day and 4,000 group µg/day doses among the three *MTHFR* genotypes (Fig. 3 and supplemental Table S2).

### Comparison of DNA methylation levels side by side in samples extracted from uncoagulated blood and coagulated blood

We found high correlation between analytical runs >2 years apart for the DNA samples re-extracted and assayed from clots (n = 20: baseline clots 1st run %MdCyt = 4.39 [SD+/−0.11]; baseline clots 2nd run %MdCyt = 4.21 [SD+/−0.13]; 1 month clot 1st run %MdCyt = 3.96 [SD+/−0.17] ; 1 month clots 2nd run %MdCyt = 3.85 [SD+/−0.23]).

To examine samples with the most consistent (1 month) and largest decreases (9 month MTHFR CC), we assayed samples (*n* = 17 participants) from the 100 µg/day at the 1 and 9 month time points, both in DNA extracted from blood and in DNA extracted from clots not previously assayed (blinded and extracted using the same protocol after the initial processing required for homogenization of the clot). We found that, at 1 month, DNA extracted from clots %MdCyt = 3.82 [SD+/−0.38] and in uncoagulated samples was %MdCyt = 4.24 [SD+/−0.22], and at 9 months in DNA extracted from clots was %MdCyt = 3.04 [SD+/−1.50] and in DNA extracted from uncoagulated blood was %MdCyt = 4.3 [SD+/−0.15], confirming the differences between the sample types and the consistency of results across samples and time.

## Discussion

This is the first population-based randomized intervention study to describe the global DNA methylation response to the initiation of and withdrawal from folic acid supplementation. In this study, no global methylation changes were associated with up to 4,000 µg/day folic acid supplementation for 6 months; when the analysis was completed using DNA extracted from uncoagulated blood. However, significant changes in DNA methylation were found in response to supplementation with folic acid and the withdrawal of supplementation in DNA extracted from coagulated blood that were time-, dose-, and genotype-dependent, suggesting that folic acid intake might impact a coagulation-mediated process.

Previous small-scale feeding studies have provided evidence that global DNA methylation decreases in response to moderate folate intake/depletion [13], [14] and increases upon subsequent repletion [14], thus an initial increase in DNA methylation was anticipated in the current study. However, no changes in DNA methylation levels were found in uncoagulated samples, which might reflect more closely circulating blood and the samples used in previous studies. One possible explanation for the lack of change observed in this study was that DNA methylation was already optimal among our participants, and would not have been expected to increase with folic acid supplementation. Alternatively, the differential results between studies possibly could be explained by the fact that previous analytical methodology used to estimate DNA methylation lacked the accuracy and precision of the LC-MS/MS method to measure the true level of genome-wide methylation [12].

Surprisingly, the data from this study also suggested that folic acid supplementation led to changes in DNA methylation levels in blood cells that were allowed to coagulate and that these changes were time-, dose-, and *MTHFR* genotype-dependent. The differential results by blood sample type were unexpected and have not been reported previously. To rule out methodological error, we replicated the quantitation of DNA methylation from blood clots by LC MS/MS up to four times, including reblinding and side-by-side analysis from extraction through analysis, with high reproducibility of results between analytical runs. We also estimated DNA methylation in the coagulated samples by using a completely different methodology (^3^H methyl acceptance), which confirmed the pattern and magnitude of change in the coagulated samples. Moreover, the analogous change in DNA methylation observed between the two assays, the decrease in %MdCty (LC-MS/MS), and the increase in unmethylated dCyt ([^3^H] methyl acceptance assay), could not be explained by changes in the quality of the DNA used in the assay. The [^3^H] methyl acceptance assay requires that the DNA be high quality and double stranded; thus, low-quality DNA would result in a decrease in [^3^H] methyl acceptance capacity (not an increase, as was observed in this study). In contrast, the LC-MS/MS assay is independent of DNA quality, as the DNA is digested to nucleosides as part of the assay. Therefore, these data supported the conclusion that the discrepancy in DNA methylation levels between blood sample types could not be attributed to methodological error.

The differential results from coagulated and uncoagulated blood were present only in association with folic acid supplement use and the large decreases in DNA methylation observed in coagulated blood after supplement withdrawal were associated strongly with the *MTHFR* genotype and consistently replicated and confirmed across time using different analytical methodology. The fact that the level of DNA methylation was the same in both sample types at baseline, with differences observed only after the initiation of folic acid supplementation, confirms the influence of folic acid supplementation and *MTHFR* genotype in DNA exposed to the clotting process. Therefore, the time-, dose-, and *MTHFR* genotype-dependent changes in DNA methylation levels in the coagulated DNA samples in response to folic acid supplementation and withdrawal cannot be considered an artifact. These data raise a series of questions related to the possible influence of coagulation and the *MTHFR* 677C→T genotype on DNA methylation when folic acid exposure is altered and sets a research agenda for these intriguing new questions to be investigated.

### Why do global methylation levels differ between coagulated and uncoagulated blood samples?

#### Differential Cell Type Distribution

In this study we utilized uncoagulated (EDTA) blood (spun down with the plasma removed) as one source of DNA. The buffy coat (the cell fraction most often used in genetic studies) should be contained completely in this fraction. Ideally, the uncoagulated and the coagulated samples (obtained through simply allowing a clot to form, then spinning down and removing the sera) should contain similar amounts and types of the nucleated blood cells (all of them), which would contribute to the DNA assayed for methylation status; however; in practice, this might not be the case. In our study, it is possible that some of the observed changes in DNA methylation in response to supplementation might have been due to differences in leukocyte cell types or specific cell subtype responses from which DNA was extracted, given that whole blood contains a mixture of cells with different turnover rates (e.g., neutrophils, lymphocytes, and monocytes) [25] whose DNA methylation patterns might differ [26]. The fractionation procedures could have left different populations of cells in the aqueous fraction (plasma or sera, respectively). To address this issue in the available samples, we compared the DNA yields from coagulated and uncoagulated blood and correlated DNA yield in the clots with methylation level. Samples yielded the expected amount of DNA based on the blood volume of the initial blood draw. There was variation in DNA yield, which is to be expected when using clots [27]; however; there was no correlation between DNA yield and global methylation levels in the samples. It seems unlikely that different cell types in the clots would have been a driving mechanism for the observed reductions in DNA methylation for a number of reasons. First, to achieve even a 14% decrease in methylation (as observed with supplementation), an extensive shift in cell type distribution within the leukocyte cell population would have been required (this is possible, but not likely). Second, achievement of a large genotype-dependent reduction (up to 90%) without a concomitant drop in DNA yield seems improbable. Additionally, many different human tissue types (e.g., heart, liver, lung, spleen, lymphocyte, brain, and thymus) among humans appear to exhibit little variation in global DNA methylation (mean = 4.52%; range = 4.28%–4.85%) [28] in contrast to ∼14% promoter of CpG sites have been reported to vary between tissues [29]. Moreover, the level of DNA methylation in monocytes from a U.S. study (4.45+/−0.12%; unpublished data) was similar to that observed for whole blood DNA at enrollment in this study in the clotted DNA samples (4.42+/−0.12%). The fact that no differences were detected in DNA methylation at baseline between the coagulated and uncoagulated samples argues against there being differences in the cell population between the two sample types.

#### Other processes associated with de-/hypo-methylation

Apoptosis is the primary consideration in any discussion of de-/hypomethylation because of the clear connection between hypomethylation and apoptosis [30], [31]. This study was not designed to assess if the samples allowed to coagulate were undergoing apoptosis. A limited number of samples run on agarose gels did not show a definitive apoptotic banding pattern or degradation that was limited to samples with decreases in assayed DNA methylation level (data not shown). However, since this study was not designed to directly assay for evidence of apoptosis, a potential influence of this process cannot be ruled out and warrants further study.

Generally, decreases in global methylation are thought to be a result of a reduced availability of substrate during DNA replication. However, it is unlikely that the hypomethylation seen in our study is replication-dependent because of the limited window of time (1–2 hours) and the large decreases reported. Active demethylation has been observed embryonically; there is a wave of demethylation as DNA methylation patterns are reset in the early embryo [32]; however, the identity the demethylase remains unknown [33]. It is possible that the hypomethylation observed in our study may be the result of active demethylation, as part or consequence of the 2 hours allowed for coagulation.

The study of coagulation has centered largely on factors in the plasma that induce coagulation. Elevated homocysteine concentration consistently has been shown to be associated with venous thrombosis; however, the nature of the association remains an active area of investigation [6], [34]. Among populations with higher folate/folic acid intake, such as those in North America and Europe, there has been no consistent association between thrombosis and *MTHFR* genotype [6], [34]. However, among populations in Asia and Latin America, the *MTHFR* T allele has been shown to be associated more consistently with an increased risk of venous thrombosis [6], [35]. It is interesting to consider the fact that the largest decreases in DNA methylation in our present study were following withdrawal of supplementation in those with the *MTHFR* C allele, which might be more resistant to coagulation. In our previous study, elevated homocysteine was associated strongly with the *MTHFR* T genotype among this population [8]. Work in human cell lines suggests that hypomethylation is a mechanism of hyperhomocysteinemia-induced cell damage [36]. Intriguingly, a small plasma proteomics study of folic acid supplementation (1.2 mg for12 weeks) found that folic acid supplementation was associated with changes in proteins involved in increased coagulation [37]. Additionally, taking a folic acid supplement during pregnancy has been shown to be associated with a reduction in hypercoagulability [38]. There is a balance between coagulation and hemorrhage, and *MTHFR* genotype and folic acid use and DNA methylation could affect this equilibrium. Future research will be needed to determine any effects on coagulation of folic acid supplementation and the termination of supplementation.

### Future research

Additional basic science research is needed to replicate our findings and provide insight into additional biological mechanisms that might explain our results. The differential results between sample types were not present at baseline, becoming evident only after initiation of the folic acid supplementation trial. Currently, studies are underway to determine if there are differences in the coagulated and uncoagulated samples of individuals who were B12-deficient and anemic who were enrolled in the study, but not eligible to participate in the intervention trial. The results from this study will help to determine if this phenomenon is limited to folic acid exposure or might be a broader effect of micronutrient status.

Although we have shown that in the uncoagulated blood there are no global methylation changes, this does not rule out changes in specific genes or genetic loci. Tests currently are under way in uncoagulated samples to determine if changes in specific genes in response to folic acid supplementation or the termination of supplementation, or both, can be detected. Additionally, controlled studies are needed to determine if these results can be replicated among populations both exposed and unexposed to folic acid, as well as other racial and ethnic and nutritional status groups.

### Implications

Although the present study does not provide definitive explanations regarding the observed influence of blood clotting, folic acid supplementation, and the *MTHFR* genotype on DNA methylation, the findings do inform future studies of DNA methylation. Specifically, these results provide new opportunities for basic researchers to identify pathways of DNA demethylation that occur during coagulation that are sensitive to *MTHFR* genotype and folic acid exposure. These data have implications for the design of future studies since clotted samples often are available as a source of genomic DNA for DNA methylation analyses in human metabolic and population surveys. DNA methylation analysis currently requires genomic DNA in relatively high quantities (several hundred ng) which can be problematic for large studies involving human participants using archival samples. The clots from serum collection are an often available and unutilized source of DNA; however, our study strongly suggested that this sample type does not show the same results as those obtained from uncoagulated blood (which might represent more closely what is present in circulating blood). The study highlights the importance of sample selection for those planning epigenetic studies. Additionally, these data provide the impetus for further investigations to clarify the observed differences in DNA methylation between coagulated and uncoagulated blood that are suggestive of an effect of folic acid on a coagulation-mediated process.

## Supporting Information

Table S1 **Global DNA methylation changes in response to folic acid supplementation and withdrawal among women of reproductive age- Coagulated Blood**.
(DOCX)

Table S2 **DNA methylation level during folic acid supplementation and withdrawal among women of reproductive age- Uncoagulated Blood**.
(DOCX)
