# Supplementary material for: Genomic DNA Methylation Changes in Response to Folic Acid Supplementation in a Population-Based Intervention Study among Women of Reproductive Age
Source: PLoS One. 2011 Dec 7;6(12):e28144. doi: 10.1371/journal.pone.0028144 (PMC3233549; doi:10.1371/journal.pone.0028144)
Supplement: Table S1 — Global DNA methylation changes in response to folic acid supplementation and withdrawal among women of reproductive age- Coagulated Blood. (DOCX) [file pone.0028144.s001.docx]

Supplemental Table 1. Global DNA methylation changes in response to folic acid supplementation and withdrawal among women of reproductive age- Coagulated Blood

|  |  | |  | |  |  |  |  |  |  | **Supplementation** | | | | | | | | **Withdrawal** | | |  |  |  |
| --- | --- | --- | --- | --- | --- | --- | --- | --- | --- | --- | --- | --- | --- | --- | --- | --- | --- | --- | --- | --- | --- | --- | --- | --- |
|  | **Folic acid**  **Dose** | | ***MTHFR***  **Genotype** | | ***n*** |  |  |  | **Enrollment** | **(95%CI)** | **1mo** | | **(95%CI)** | | **3mo** | **(95%CI)** | **6mo** | **(95%CI)** | **9mo** | **(95%CI)** | |  |  |  |
| **overall ^a^** |  | |  | | **135** | **Estimated mean %MdCyt** |  |  | 4.42 | (4.41-4.43) | 3.78 ^b^ | | (3.73-3.82) | | 3.83 ^b^ | (3.78--3.89) | 3.92 ^b^ | (3.83-4.02) | 3.01 ^b^ | (2.86-3.16) | |  |  |  |
|  |  | |  | |  | **% change from enrollment** |  |  |  |  | -14.5 | | (-20.5- -7.7) | | -13.3 | (-19.8- -7.0) | -11.2 | (-17.4- -4.2) | -32.0 | (-35.7--25.5) | |  |  |  |
|  |  | |  | |  | **% change following withdrawal** | |  |  |  |  | |  | |  |  |  |  | -23.4 | (-27.7- -16.3) | |  |  |  |
|  |  | |  | |  |  |  |  |  |  |  | |  | |  |  |  |  |  |  |  |  |  |  |
|  | **100 µg/d** | |  | | **45** | **Estimated mean %MdCyt** |  |  | 4.44 | (4.42-4.46) | 3.82 ^c^ | | (3.75-3.90) | | 3.93 ^c^ | (3.84-4.03) | 4.00 ^d^ | (3.84-4.16) | 2.71 ^c^ | (2.49-2.94) | |  |  |  |
|  |  | |  | |  | **% change from enrollment** | |  |  |  | -14.0 | | (-23.9- -3.1) | | -11.5 | (-22.7- -1.8) | -10.0 | (-20.5-1.1) | -39.1 | (-44.2--29.2) | |  |  |  |
|  |  | |  | |  | **% change following withdrawal** | |  |  |  |  | |  | |  |  |  |  | -32.3 | (-37.8—21,0) | |  |  |  |
|  | **400 µg/d** | |  | | **45** | **Estimated means %MdCyt** |  |  | 4.41 | (4.39-4.42) | 3.80 ^c^ | | (3.72-3.88) | | 3.89 ^c^ | (3.79-3.99) | 3.88 ^c^ | (3.71--4.05) | 3.43 ^c n^ | (3.14-3.75) | |  |  |  |
|  |  | |  | |  | **% change from enrollment** | |  |  |  | -13.7 | | (-23.7- -2.0) | | -11.7 | (-22.1- 0.1) | -12.0 | (-21.8- 0.1) | -22.1 | (-31.1- -11.6) | |  |  |  |
|  |  | |  | |  | **% change following withdrawal** | |  |  |  |  | |  | |  |  |  |  | -11.5 | (-22.0- -0.2) | |  |  |  |
|  | **4000 µg/d** | | | | **45** | **Estimated mean %MdCyt** |  |  | 4.41 | (4.40-4.43) | 3.71 ^c^ | | (3.63-3.68) | | 3.58 ^c l^ | (3.78-3.78) | 3.90 ^c^ | (3.73-4.08) | 2.93 ^c^ | (2.67-3.21) | |  |  |  |
|  |  | |  | |  | **% change from enrollment** | |  |  |  | -15.9 | | (-26.4- -2.8) | | -18.9 | (-27.1- -3.8) | -11.6 | (-22.7-2.0) | -33.7 | (-41.1--22.3) | |  |  |  |
|  |  | |  | |  | **% change following withdrawal** | |  |  |  |  | |  | |  |  |  |  | -25.0 | (-33.6- -12.7) | |  |  |  |
|  |  | |  | |  |  |  |  |  |  |  | |  | |  |  |  |  |  |  |  |  |  |  |
|  |  | | **CC** | | **43** | **Estimated mean %MdCyt** |  |  | 4.45 | (4.42-4.47) | 3.64 ^e^ | | (3.54-3.74) | | 4.00 ^e^ | (3.87-4.13) | 3.75 ^e^ | (3.55-3.97) | 2.19 ^e^ | (1.95-2.46) | |  |  |  |
|  |  | |  | |  | **% change from enrollment** | |  |  |  | -18.1 | | (-30.2- -2.8) | | -10.1 | (-24.6-4.8) | -15.6 | (-28.1- -0.4) | -50.7 | (-56.3--39.2) | |  |  |  |
|  |  | |  | |  | **% change following withdrawal** | |  |  |  |  | |  | |  |  |  |  | -41.7 | (-48.2- -28.3) | |  |  |  |
|  |  | | **CT** | | **45** | **Estimated means %MdCyt** |  |  | 4.43 | (4.42-4.44) | 3.83 ^e k^ | | (3.77-3.89) | | 3.75 ^e^ | (3.68-3.82) | 3.98 ^e^ | (3.85-4.11) | 3.18 ^e o^ | (2.98-3.4) |  |  |  |  |
|  |  | |  | |  | **% change from enrollment** | |  |  |  | -13.5 | | (-21.2- -4.2) | | -15.3 | (-23.3- -6.6) | -10.1 | (-18.3- -0.4) | -28.2 | (-33.6--19.2) | |  |  |  |
|  |  | |  | |  | **% change following withdrawal** | |  |  |  |  | |  | |  |  |  |  | -20.1 | (-26.4- -10.4) | |  |  |  |
|  |  | | **TT** | | **48** | **Estimated mean %MdCyt** |  |  | 4.39 **^j^** | (4.37-4.40) | 3.87 ^e k^ | | (3.79-3.95) | | 3.75 ^e^ | (3.66-3.84) | 4.04 ^e^ | (3.89-4.21) | 3.90 ^f o^ | (3.59-4.23) | |  |  |  |
|  |  | |  | |  | **% change from enrollment** | |  |  |  | -11.8 | | (-21.5- -1.7) | | -14.5 | (-23.5- -4.3) | -7.8 | (-17.5- 3.2) | -11.1 | (-21.4- -1.9) | |  |  |  |
|  |  | |  | |  | **% change following withdrawal** | |  |  |  |  | |  | |  |  |  |  | -3.6 | (-14.8- 6.3) | |  |  |  |
|  |  | |  | |  |  |  |  |  |  |  | |  | |  |  |  |  |  |  |  |  |  |  |
|  | **100 µg/d** | | **CC** | | **15** | **Estimated mean %MdCyt** |  |  | 4.44 | (4.40- 4.47) | 3.87 ^g^ | | (3.73-4.02) | | 3.99 ^g^ | (3.81-4.17) | 3.87 ^g^ | (3.58-4.18) | 1.64 ^g^ | (1.4-1.92) |  |  |  |  |
|  |  | |  | |  | **% change from enrollment** | |  |  |  | -12.8 | | (-32.7-10.7) | | -10.1 | (-32.4-10.3) | -12.9 | (-33.1-9.2) | -63.1 | (-70.0- -51.0) | |  |  |  |
|  |  | |  | |  | **% change following withdrawal** | |  |  |  |  | |  | |  |  |  |  | -57.7 | (-64.9- -42.7) | |  |  |  |
|  |  | | **CT** | | **15** | **Estimated means %MdCyt** |  |  | 4.48 | (4.46-4.51) | 3.81 ^g^ | | (3.7-3.92) | | 3.90 ^f^ | (3.76-4.04) | 3.74 ^h^ | (3.51-3.97) | 2.77 ^g p^ | (2.44-3.14) | |  |  |  |
|  |  | |  | |  | **% change from enrollment** | |  |  |  | -15.0 | | (-31.6-5.1) | | -13.0 | (-31.1-5.9) | -16.6 | (-33.1-3.3) | -38.3 | (-47.6- -19.5) | |  |  |  |
|  |  | |  | |  | **% change following withdrawal** | |  |  |  |  | |  | |  |  |  |  | -25.9 | (-34.8- -6.2) | |  |  |  |
|  |  | | **TT** | | **15** | **Estimated mean %MdCyt** |  |  | 4.41 | (4.38-4.44) | 3.79 ^g^ | | (3.66-3.92) | | 3.91 ^g^ | (3.75-4.07) | 4.43 | (4.13-4.74) | 4.37 ^p^ | (3.8-5.04) |  |  |  |  |
|  |  | |  | |  | **% change from enrollment** | |  |  |  | -14.1 | | (-32.4-5.2) | | -11.3 | (-30.1-8.8) | 0.4 | (-20.9-23.0) | -0.8 | (-23.0- 20.6) | |  |  |  |
|  |  | |  | |  | **% change following withdrawal** | |  |  |  |  | |  | |  |  |  |  | -1.2 | (-18.6-18.9) | |  |  |  |
|  | **400 µg/d** | | **CC** | | **15** | **Estimated mean %MdCyt** |  |  | 4.40 | (4.36-4.44) | 3.60 ^i^ | | (3.43-3.78) | | 4.03 ^g^ | (3.81-4.27) | 3.66 ^i^ | (3.32-4.04) | 3.02 ^g p^ | (2.47-3.69) | |  |  |  |
|  |  | |  | |  | **% change from enrollment** | |  |  |  | -18.14 | | (-36.98-9.27) | | -8.3 | (-30.71-20.14) | -16.72 | (-35.41-10.97) | -31.4 | (-46.2- -6.6) | |  |  |  |
|  |  | |  | |  | **% change following withdrawal** | |  |  |  |  | |  | |  |  |  |  | -17.5 | (-36.1- 9.8) | |  |  |  |
|  |  | | **CT** | | **15** | **Estimated means %MdCyt** |  |  | 4.42 | (4.40-4.44) | 3.84 ^g^ | | (3.75-3.95) | | 3.80 ^g^ | (3.69-3.92) | 4.02 ^g^ | (3.81-4.24) | 3.17 ^g p^ | (2.85-3.53) | |  |  |  |
|  |  | |  | |  | **% change from enrollment** | |  |  |  | -13.0 | | (-30.2-10.0) | | -14.0 | (-30.9-8.9) | -9.1 | (-26.7-15.6) | -28.2 | (-42.5- -9.3) | |  |  |  |
|  |  | |  | |  | **% change following withdrawal** | |  |  |  |  | |  | |  |  |  |  | -21.03 | (-33.5- -7.4) | |  |  |  |
|  |  | | **TT** | | **15** | **Estimated mean %MdCyt** |  |  | 4.38 | (4.36-4.41) | 3.96 ^g^ | | (3.84-4.1) | | 3.84 ^g^ | (3.69-3.99) | 3.95 ^i^ | (3.7-4.23) | 4.21 ^p^ | (3.67-4.84) | |  |  |  |
|  |  | |  | |  | **% change from enrollment** | |  |  |  | -9.6 | | (-28.8-14.8) | | -12.4 | (-30.6-12.0) | -9.8 | (-28.8-14.2) | -3.8 | (-25.6- 19.4) | |  |  |  |
|  |  | |  | |  | **% change following withdrawal** | |  |  |  |  | |  | |  |  |  |  | 6.6 | (-13.4- 26.1) | |  |  |  |
|  | **4000 µg/d** | | **CC** | | **12** | **Estimated mean %MdCyt** |  |  | 4.49 | (4.44-4.53) | 3.47 ^g^ | | (3.29-3.66) | | 3.97 ^g^ | (3.74-3.35) | 4.17 ^g^ | (3.37-4.18i) | 2.13 ^g^ | (1.7-2.66) |  |  |  |  |
|  |  | |  | |  | **% change from enrollment** | |  |  |  | -22.7 | | (-43.8-8.4) | | -11.6 | (-35.8-23.7) | -7.1 | (-39.8-16.1) | -52.6 | (-63.7- -30.0) | |  |  |  |
|  |  | |  | |  | **% change following withdrawal** | |  |  |  |  | |  | |  |  |  |  | -48.6 | (-56.3- -16.8) | |  |  |  |
|  |  | | **CT** | | **15** | **Estimated means %MdCyt** |  |  | 4.39 | (4.36-4.41) | 3.84 ^g^ | | (3.73-3.94) | | 3.56 ^g^ | (3.45-3.68) | 4.20 ^h^ | (3.98-4.43) | 3.67 ^g p^ | (3.28-4.1) |  |  |  |  |
|  |  | |  | |  | **% change from enrollment** | |  |  |  | -12.5 | | (-33.7-12.6) | | -18.8 | (-38.5-4.3) | -4.3 | (-27.9-22.4) | -16.4 | (-37.1- 6.8) | |  |  |  |
|  |  | |  | |  | **% change following withdrawal** | |  |  |  |  | |  | |  |  |  |  | -12.7 | (-25.7- 2.5) | |  |  |  |
|  |  | | **TT** | | **18** | **Estimated mean %MdCyt** |  |  | 4.37 | (4.34-4.40) | 3.86 ^g^ | | (3.73-3.99) | | 3.52 ^g^ | (3.38-3.67) | 3.78 ^i^ | (3.53-4.05) | 3.22 ^h p^ | (2.79-3.71) | |  |  |  |
|  |  | |  | |  | **% change from enrollment** | |  |  |  | -11.7 | | (-35.9-23.7) | | -19.5 | (-39.8-16.1) | -13.5 | (-35.4-12.5) | -26.4 | (-45.8- -5.6) | |  |  |  |
|  |  | |  | |  | **% change following withdrawal** | |  |  |  |  | |  | |  |  |  |  | -14.8 | (-31.1- 2.1) | |  |  |  |
|  |  |  | |  |  |  |  | |  |  |  |  | |  |  |  |  |  |  |  |  |  | |  |

^a^ All estimated mean %MdCyt and 95% CI were generated from a general linear model of repeated measure model (GLMRM) containing; folic acid dose, *MTHFR* genotype, age, BMI, enrollment RBC and time (0,1,3,6,9 months data) weighted to adjust for sampling. The model shows a significant interaction of folic acid dose and *MTHFR* genotype (P< 0.001).

^b^  P < 0.001 compared to enrollment in full GLMRM model (folic acid dose, *MTHFR* genotype, age, BMI, enrollment RBC and time)

^c^  P < 0.001 compared to enrollment in GLMRM model stratified by folic acid dose (*MTHFR* genotype, age, BMI, enrollment RBC and time)

^d^  P < 0.01 compared to enrollment in GLM RM model stratified by folic acid dose (*MTHFR* genotype, age, BMI, enrollment RBC and time)

^e^  P < 0.001 compared to enrollment in GLM RM model stratified by *MTHFR* genotype (folic acid dose, age, BMI, enrollment RBC and time)

^f^  P < 0.01 compared to enrollment in GLM RM model stratified by *MTHFR* genotype (folic acid dose, age, BMI, enrollment RBC and time)

^g^  P < 0.001 compared to enrollment in GLM RM model stratified by folic acid dose and *MTHFR* genotype (age, BMI, enrollment RBC and time)

^h^  P < 0.05 compared to enrollment in GLM RM model stratified by folic acid dose and *MTHFR* genotype (age, BMI, enrollment RBC and time)

^i^  P < 0.01 compared to enrollment in GLM RM model stratified by folic acid dose and *MTHFR* genotype (age, BMI, enrollment RBC and time)

**^j^**  P < 0.01 CC vs. TT at enrollment GLM model adjusted (folic acid dose, age, BMI and baseline RBC)

**^k^** P < 0.01 CC vs. TT and CC vs. TT at 1 month GLM model (folic acid dose, age, BMI and baseline RBC)

**^l^** P < 0.01 100µg vs. 4,000µg at 3 month GLM model (folic acid dose, age, BMI and baseline RBC)

**^m^** P < 0.01 CC vs. TT and CC vs. TT at 3 month GLM model (folic acid dose, age, BMI and baseline RBC)

**^o^** P < 0.001 CC vs. TT and CC vs. TT at 9 month GLM model (folic acid dose, age, BMI and baseline RBC)

**^p^** P < 0.001 100µg CC vs. 100µg CT, 100µg TT, 400µg CC, 400µg CT, 400µg TT, 4000µg CT, 4000µg TT at 9 month GLM model (folic acid dose, age, BMI and baseline RBC)
