# Supplementary material for: Genomic DNA Methylation Changes in Response to Folic Acid Supplementation in a Population-Based Intervention Study among Women of Reproductive Age
Source: PLoS One. 2011 Dec 7;6(12):e28144. doi: 10.1371/journal.pone.0028144 (PMC3233549; doi:10.1371/journal.pone.0028144)
Supplement: Table S2 — DNA methylation level during folic acid supplementation and withdrawal among women of reproductive age- Uncoagulated Blood. (DOCX) [file pone.0028144.s002.docx]

Supplemental Table 2. DNA methylation level during folic acid supplementation and withdrawal among women of reproductive age- Uncoagulated Blood

|  |  |  |  |  |  | | **Supplementation** | | | | | | **Withdrawal** | |
| --- | --- | --- | --- | --- | --- | --- | --- | --- | --- | --- | --- | --- | --- | --- |
|  | **Folic acid**  **dose** | ***MTHFR***  **genotype** | ***n*** | **Enrollment**  **%MdCyt** | **(95% CI)** | **1 mo**  **%MdCyt** | | **(95% CI)** | **3 mo**  **%MdCyt** | **(95% CI)** | **6 mo**  **%MdCyt** | **(95% CI)** | **9 mo**  **%MdCyt** | **(95% CI)** |
|  |  |  |  |  |  |  | |  |  |  |  |  |  |  |
| **Overall ^a^** |  |  | 59 | 4.45 | (4.42-4.49) | 4.43 | | (4.42-4.45) | 4.49 | (4.47-4.52) | 4.47 | (4.45-4.50) | 4.47 | (4.44-4.49) |
|  |  |  |  |  |  |  | |  |  |  |  |  |  |  |
|  | **400µg/d** |  | 30 | 4.44 | (4.39-4.49) | 4.44 | | (4.42-4.47) | 4.49 | (4.45-4.52) | 4.48 | (4.45-4.51) | 4.46 | (4.42-4.50) |
|  | **4000µg/d** |  | 29 | 4.46 | (4.41-4.52) | 4.43 | | (4.40-4.45) | 4.50 | (4.46-4.54) | 4.47 | (4.44-4.50) | 4.47 | (4.44-4.51) |
|  |  |  |  |  |  |  | |  |  |  |  |  |  |  |
|  |  | **CC** | 20 | 4.46 | (4.38-4.54) | 4.43 | | (4.39-4.47) | 4.51 | (4.46-4.57) | 4.47 | (4.42-4.52) | 4.46 | (4.41-4.52) |
|  |  | **CT** | 17 | 4.46 | (4.41-4.51) | 4.45 | | (4.42-4.47) | 4.46 | (4.43-4.50) | 4.48 | (4.45-4.51) | 4.46 | (4.43-4.50) |
|  |  | **TT** | 22 | 4.44 | (4.38-4.50) | 4.43 | | (4.40-4.46) | 4.50 | (4.45-4.54) | 4.47 | (4.43-4.51) | 4.47 | (4.43-4.52) |
|  |  |  |  |  |  |  | |  |  |  |  |  |  |  |
|  | **400µg/d** | **CC** | 10 | 4.46 | (4.36-4.55) | 4.45 | | (4.4-4.49) | 4.46 | (4.39-4.53) | 4.46 | (4.4-4.51) | 4.44 | (4.37-4.50) |
|  |  | **CT** | 7 | 4.41 | (4.32-4.50) | 4.46 | | (4.42-4.50) | 4.54 | (4.47-4.60) | 4.48 | (4.43-4.53) | 4.45 | (4.38-4.51) |
|  |  | **TT** | 13 | 4.47 | (4.39-4.55) | 4.42 | | (4.38-4.46) | 4.47 | (4.41-4.52) | 4.50 | (4.46-4.55) | 4.50 | (4.44-4.55) |
|  |  |  |  |  |  |  | |  |  |  |  |  |  |  |
|  | **4000µg/d** | **CC** | 10 | 4.46 | (4.34-4.59) | 4.41 | | (4.35-4.47) | 4.57 | (4.48-4.66) | 4.48 | (4.41-4.55) | 4.49 | (4.40-4.58) |
|  |  | **CT** | 10 | 4.51 | (4.45-4.58) | 4.44 | | (4.40-4.47) | 4.39 | (4.34-4.44) | 4.49 | (4.45-4.53) | 4.48 | (4.44-4.53) |
|  |  | **TT** | 9 | 4.42 | (4.32-4.52) | 4.43 | | (4.38-4.48) | 4.53 | (4.46-4.60) | 4.43 | (4.38-4.49) | 4.45 | (4.38-4.52) |
|  |  |  |  |  |  |  | |  |  |  |  |  |  |  |

^a^ Estimated mean %MdCyt and 95% CI were generated from a general linear model of repeated measure model (GLMRM) containing; folic acid dose, *MTHFR* genotype, age, BMI and time (0,1,3,6,9 months data) weighted to adjust for sampling. GLMRM modeling shows no significant effect of the *MTHFR* genotype or folic acid dose or an interaction of dose and genotype (all P >0.8). All pairwise comparisons between enrollment and months of supplementation and withdrawal were non-significant. All subjects included in this table are a subset of coagulated results in Table 2. All change from baseline methylation levels throughout supplementation and termination of supplementation are < +/- 3% which is within the variability of the LC-MS/MS assay.
